# Whole plant based treatment of hypercholesterolemia with *Crataegus laevigata* in a zebrafish model

**DOI:** 10.1186/1472-6882-12-105

**Published:** 2012-07-23

**Authors:** Robert M Littleton, Matthew Miller, Jay R Hove

**Affiliations:** 1 Department of Molecular and Cellular Physiology, University of Cincinnati College of Medicine, Cincinnati, OH, USA; 2 Molecular and Developmental Biology, Cincinnati Children's Hospital Medical Center, Cincinnati, OH, USA

## Abstract

**Background:**

Consumers are increasingly turning to plant-based complementary and alternative medicines to treat hypercholesterolemia. Many of these treatments are untested and their efficacy is unknown. This multitude of potential remedies necessitates a model system amenable to testing large numbers of organisms that maintains similarity to humans in both mode of drug administration and overall physiology. Here we develop the larval zebrafish (4–30 days post fertilization) as a vertebrate model of dietary plant-based treatment of hypercholesterolemia and test the effects of *Crataegus laevigata* in this model*.*

**Methods:**

Larval zebrafish were fed high cholesterol diets infused with fluorescent sterols and phytomedicines. Plants were ground with mortar and pestle into a fine powder before addition to food. Fluorescent sterols were utilized to optically quantify relative difference in intravascular cholesterol levels between groups of fish. We utilized the Zeiss 7-Live Duo high-speed confocal platform in order to both quantify intravascular sterol fluorescence and to capture video of the heart beat for determination of cardiac output.

**Results:**

In this investigation we developed and utilized a larval zebrafish model to investigate dietary plant-based intervention of the pathophysiology of hypercholesterolemia. We found BODIPY-cholesterol effectively labels diet-introduced intravascular cholesterol levels (P < 0.05, Student’s *t*-test). We also established that zebrafish cardiac output declines as cholesterol dose increases (difference between 0.1% and 8% (w/w) high cholesterol diet-treated cardiac output significant at P < 0.05, 1-way ANOVA). Using this model, we found hawthorn leaves and flowers significantly reduce intravascular cholesterol levels (P < 0.05, 1-way ANOVA) and interact with cholesterol to impact cardiac output in hypercholesterolemic fish (2-way ANOVA, P < 0.05 for interaction effect).

**Conclusions:**

The results of this study demonstrate that the larval zebrafish has the potential to become a powerful model to test plant based dietary intervention of hypercholesterolemia. Using this model we have shown that hawthorn leaves and flowers have the potential to affect cardiac output as well as intravascular cholesterol levels. Further, our observation that hawthorn leaves and flowers interact with cholesterol to impact cardiac output indicates that the physiological effects of hawthorn may depend on diet.

## Background

Adults in the United States spent $33.9 billion out-of-pocket on complementary and alternative medicines (CAMs) in 2007, nearly half of which went toward purchasing nonvitamin, nonmineral natural products (NVNMNPs) [1].

One of the most common uses of NVNMNPs is for the management of blood cholesterol (CH) levels. Despite a poor understanding of the efficacy and effects of these treatments consumers can find dozens of over-the-counter plant-based CH remedies at local grocery stores or pharmacies. Not surprisingly, practitioners of Western medicine frequently dismiss phytomedical options due to the lack of experimentally derived data on their effects and modes of action. One reason for this empirical deficit is that each plant has numerous potential effects and for any prescribed ailment there are many candidate plant-based treatments. Further complicating the matter, the effects of these treatments may be subtler than those of purified Western pharmaceuticals, all of which necessitates testing large numbers of organisms. Mammalian model systems offer a potential solution to this problem however, while offering the benefit of a close phylogenetic proximity to humans, they are expensive to house and to maintain. Alternatively, cell culture-based models of disease offer the advantage of significantly higher-throughput testing at substantially lower cost. Unfortunately, *in vitro* experiments can recapitulate neither the biological complexity nor the physiochemical connectivity of an intact vertebrate system. As a result, the extent to which these data represent the patterns and processes in the actual human disease condition is somewhat limited.

The high-fecundity, rapid development, low husbandry costs and optical clarity of their larvae have contributed to the zebrafish's emergence as a premier vertebrate model in biomedicine [2]. Several studies have demonstrated that zebrafish digestive physiology and lipid metabolism are very similar to that of humans and that treatment of zebrafish with antihyperlipidemic drugs elicits similar responses to their mammalian counterparts [3-6]. Recent work has also shown that zebrafish exhibit similarities to human lipid-related pathologies including increased vascular permeability and thickening, increased levels of total CH, LDL and oxidized cholesteryl esters [5,7]. Further, blood serum lipid levels in adult zebrafish can be reduced by treatment with herbal extracts of laurel, turmeric, cinnamon and clove [8,9]. These data are promising, and combined with the observation that the cardiodynamic response of embryonic zebrafish to many pharmacological agents is similar to those observed in mammals [10-15] makes it a potentially powerful nontraditional model for studying the physiological effects of a high cholesterol diet (HCD) and its treatment through diet-based phytomedical interventions.

Western pharmaceutical medicine is largely based on a reductionist paradigm of both disease and its treatment where an emphasis is placed upon the molecular mechanics of single purified/synthesized molecules and their influence on specific body receptors. A more holistic view of disease and its treatment, where whole plant products are administered, may allow for treatment of not only one characteristic of the disease but potentially several of its peripheral aspects as well [16,17]. In contrast to the inherent complexity involved in analyzing the molecular interactions and specific physiological effects of each potentially active component in a phytotherapy, we propose to initially focus only on holistically relevant bioprocesses. In this study we measure both CH and cardiac output (CO) as indicators of overall cardiovascular health under conditions of diet-induced hypercholesterolemia. We suggest that the ability of any treatment (CAM or traditional) to positively alter these metrics indicates a potent therapeutic potential.

The hawthorn (*Crataegus sp*.) plant has been utilized for thousands of years to treat a variety of illnesses. In Eastern Traditional medical systems, hawthorn is commonly used to alleviate digestive ailments and poor circulation. In Western medicine, particularly in Europe, the hawthorn plant is utilized to treat cardiovascular maladies such as cardiac failure for which it is certified to treat New York Heart Association Type II heart failure [18]. Its purported cardiotonic properties include inotropic, chronotropic [19] and vasodilatory effects [20], but the evidence for these effects comes largely from isolated organ and cell culture-based studies where the overall whole-animal physiological impact cannot readily be assessed. Hawthorn is also purported to possess antihypercholesterolemic properties. A recent study by Dalli *et al.*[21] revealed that leaf and flower extracts of *Crataegus laevigata* (HLF) appeared to lower LDL CH levels in diabetic patients with coronary heart disease, although their results were not statistically significant. Similarly, studies in both rats and rabbits have demonstrated hypolipidemic effects of hawthorn berries (HB) [22,23]. Potential antihypercholesterolemic effects and cardiotonic activity suggests hawthorn may have substantial utility as a treatment for the multi-faceted pathophysiology of lipid-based diseases.

Here we present data demonstrating the utility of the zebrafish model in empirically assessing the therapeutic potential of CAMs by testing whole plant treatments on animals with diet-induced hypercholesterolemia. This holistic approach to treatment recognizes that putative synergistic actions of components of the plant may have benefits different from those conferred by each individual molecule [16,17]. We begin by experimentally identifying the best optical marker for quantifying diet-induced hypercholesterolemia in the larval zebrafish. We then use our animal model system to assess the effects of hawthorn on the cardiovascular pathophysiology of hypercholesterolemia and finally, we test the ability of a commonly used CAM, hawthorn, to influence CO in both healthy and diseased animals.

## Methods

### Zebrafish care

Adult zebrafish were housed in the Cincinnati Children’s Hospital Medical Center (CCHMC) - University of Cincinnati (UC) zebrafish facility. Embryos were generated for this study from in-house lines of adult fish being bred, raised, and cared for according to established procedures [24]. Specific ethical approval was given for all zebrafish husbandry and experimental procedures performed at CCHMC and UC by the Institutional Animal Care and Use Committee (IACUC) protocol # 1D03020. Water conditions in our facility (pH = 7.1-7.4; temperature = 26.5-28.5°C; conductivity = 490–530 μS; and dissolved oxygen concentration = 5.0-7.5 mg L^-1^ were rigorously maintained through real-time computerized monitoring and dosing. For this study we crossed transgenic *TG*(*kdrl:mCherry*) zebrafish with mCherry fluorescent protein driven by the cardiovascular specific kdrl promoter with a *casper* line containing a melanocyte/iridophore mutation [25]. The resulting double transgenic animals *TG(Kdrl:mCherry)/Casper* express red fluorescence in the vascular walls and are optically transparent through adulthood.

### Food preparation

An HCD was created by mixing AZOO artificial *Artemia* with cholesterol (Invitrogen) in diethyl ether (Sigma). 5–80 μg/g_food_ 23-(dipyrrometheneboron difluoride)-24-norcholesterol (BOD-CH. TopFluor, Avanti Polar Lipids) and 5–80 μg/g_food_ 22-(N(−7-nitrobenz-2-oxa-1,3-diazol-4-yl)amino)-23,24-bisnor-5-cholen-3β-ol (NBD-CH. Invitrogen) were added to food in both control and HCD after Stoletov *et al.*, 2009 [4]. Hawthorn (*Crataegus laevigata*) leaves and flowers, hawthorn berries, and whole goldenrod (*Solidago virgaurea*) were obtained from Starwest Botanicals (Rancho Cordova, California). Goldenrod was utilized as a negative control to assure that plant-infused food itself did not lead to decreased fluorescent output. Herbal treatments were mixed into food at 6% and 12% (w/w) with cholesterol and diethyl ether. In the sterol fluorophore-based experiments, this combination was allowed to dry after which BOD-CH was added.

### Measurement of fluorophore levels

From 4–8 days post fertilization (dpf) zebrafish embryos were fed paramecia and housed in 1 L breeding tanks (Aquatic Habitats). Larval fish (8–21 dpf) were transferred to the main system and fed approximately 2 mg of food labeled with fluorescent sterol daily. Food weight for each treatment was measured every other day. Fish were not given normally scheduled feedings prior to imaging. Fish were anesthetized in 125-150 mg L^-1^ MS-222 (tricaine) and mounted in 1.2% agarose in glass bottomed viewing slides. Confocal imaging was performed using a LSM 710 platform within the Live Microscopy Core at the University of Cincinnati. Nine mid-sagittal Z-plane cross-sections were taken for each fish at a magnification of 20x. BOD-CH and NBD-CH were excited with a laser at 488 nm, and 458 nm λs respectively and emission captured between 503-580 nm, and 500-558 nm λs respectively. Mean fluorescence intensity of each individual Z-stack was calculated by automated analysis of collated images from collected mid-sagittal Z-stacks using Improvision Volocity (Perkin Elmer).

### Ezetimibe treatment method

Method adapted from a protocol kindly provided by Steven Farber, PhD. At 4 dpf embryos were incubated in 96-well plates with 100 μL solution per well. Solutions were made in 1 mL preparations and divided into 10 wells with 2 fish in each well. The ezetimibe treatment consisted of 2.5% v/v egg yolk in tank water with 50 μM ezetimibe (Ryan Scientific) (from a stock concentration of 10 mg/mL in DMSO), and 2.5 μg/mL BOD-CH (8 μL stock at a concentration of 0.3125 μg/μL in DMSO). The control solution was exactly the same except without ezetimibe. After incubation for 4 hours, fish were extracted from the treatments and allowed to swim in tank water for 6 hours. Fish were then imaged and data was analyzed as above.

### Percent diet uptake

For each treatment, 5 fish at 19 dpf were placed in a 100 mL beaker filled with 50 mL of tank water. The treatments were plain food (control), 4%CH-treated food, and 4%CH + 6%HLF-treated food. 10 mg of each diet was fed to each treatment group (2 mg per fish). After 3 hours feeding, fish were carefully removed from water so as not to disturb remaining food. Water was poured through previously weighed Whatman filter paper. Whatman paper with food was allowed to dry and weighed again. The final weight of the dry Watman paper with food was subtracted from the initial weight to obtain the total weight of food remaining after feeding. This value was then subtracted from the initial amount of food fed to each group, which gave the total amount of food eaten. To determine the percent intake, the total amount of food eaten was divided by the initial amount of food administered. This experiment was repeated 3 times.

### Cardiac output determination

*TG(Kdrl:mCherry)/Casper* animals were anesthetized in tricaine and mounted in agarose as described above. Feeding and care regimen were also the same except fish were fed experimental diet until 27 dpf. Fish were mounted in an upright fashion (aligned vertically) with the dorsoventral axis in vertical orientation to view the ventricular chamber from the ventral side of the fish. For these experiments, an inverted Zeiss 7-Live imaging system was utilized to capture high-speed confocal images. Images were captured at an excitation λ of 560 nm and emission was gathered with a long-pass 560 nm filter at 23 frames per second for 5 seconds. Cardiac output was calculated by measuring the equatorial (a) and polar radii (b) of the ventricular chamber assuming the shape of the ventricle approximates a prolate spheroid with the equation: V_ventricle_ = 43 × πa^2^b, where V_ventricle_ is the volume of the ventricle. V_diastole_ – V_systole_ = ΔV, represents stroke volume. ΔV × HR = CO where HR is heart rate and CO is cardiac output.

### Statistics

Regression analyses, 1-way ANOVAs, 2-way ANOVAs and student’s t-tests were performed on SigmaStat software. Holm-Sidak post-hoc multiple comparison procedure was implemented for all ANOVA tests where significant differences were observed. In all experiments, data from fish with morphological defects were not included in statistical analyses.

## Results

### BODIPY-cholesterol is a better marker of intravascular cholesterol accumulation than NBD-cholesterol

**Figure 1:**
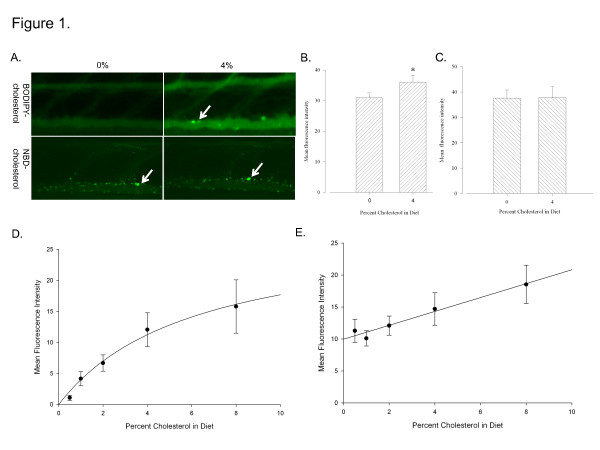
**BODIPY-cholesterol (BOD-CH) *vs.* NBD-cholesterol (NBD-CH). A**. Green is BOD-CH and NBD-CH fluorescence. Arrows point to fluorescent deposits in vasculature. Top panel: 0% HCD + 10 μg/g BOD-CH *vs.* 4% HCD + 10 μg/g BOD-CH. Bottom panel: 0% HCD + 10 μg/g NBD-CH *vs.* 4% HCD + 10 μg/g NBD-CH **B**. 0% HCD + 10 μg/g BODIPY-CH shows significantly less fluorescent output than 4% HCD + 10 μg/g BOD-CH (*P < 0.05, Student’s *t*-test. n = 12 in each group) **C**. 4% HCD + 10 μg/g NBD-CH is not different from 0% HCD + 10 μg/g NBD-CH **D**. Dose response: BOD-CH fluorescence *vs.* percent cholesterol in diet. 0.5-8% HCD + 5-80ug/g BOD-CH (R = 0.912, R^2^ = 0.83). 1-way ANOVA with Holm-Sidak multiple comparison indicates statistically significant difference between each group and every other group (P < 0.05, n = 11-12 in each group) **E**. Dose response: NBD-CH fluorescence *vs.* percent cholesterol in diet (R = 0.811, R^2^ = 0.657). Each group is significantly different from all others (P < 0.05, 1-way ANOVA, Holm-Sidak multiple comparison. n = 10-12 in each group) except 0.5, 1 and 2% which are not significantly different from each other in any combination.

To find a reliable optical marker of diet-induced intravascular cholesterol levels, we compared fluorescent sterol probes BOD-CH and NBD-CH. Our initial experiment tested the ability of each fluorescent sterol to label intravascular CH levels due to dietary intake. We fed diets with equal concentrations of fluorophore added to 0% and 4% HCD-fed groups. We also confirmed similar development of 0% and 4% HCD-fed fish by measuring body length (see Additional file: 1 Figure S1). Fish in the 4% HCD + BOD-CH treated group showed increased fluorescence compared to controls while NBD-CH treated fish demonstrated almost no difference between 4% and 0% HCD-fed groups (Figure1A and 1B). To determine the optimal CH concentration for future fluorophore-based experiments, we tested the relationship between CH and fluorophore uptake into the vasculature as each were proportionally increased, *e.g.* 0.5% CH + 5 μg/g fluorophore was fed in the lowest level treatment group and 8% HCD + 80 μg/g fluorophore was fed in the highest dose treatment group. NBD-CH demonstrated a linear increase in fluorescence with CH dose, but the rate of increase in fluorescence with dose of cholesterol was much less than that of BOD-CH (Figure1E). BOD-CH treated fish, under the same experimental conditions, showed a much stronger relationship between BODIPY fluorescence and cholesterol dose between 0.5-4% HCD. In treatments with an HCD ranging from 4-8% this relationship declined markedly (Figure1D) and so we chose 4% HCD + BOD-CH as optimum to test the effects of HB and HLF on intravascular CH levels.

### Dietary administration of hawthorn leaves and flowers improves intravascular cholesterol levels in zebrafish model of hypercholesterolemia

**Figure 2:**
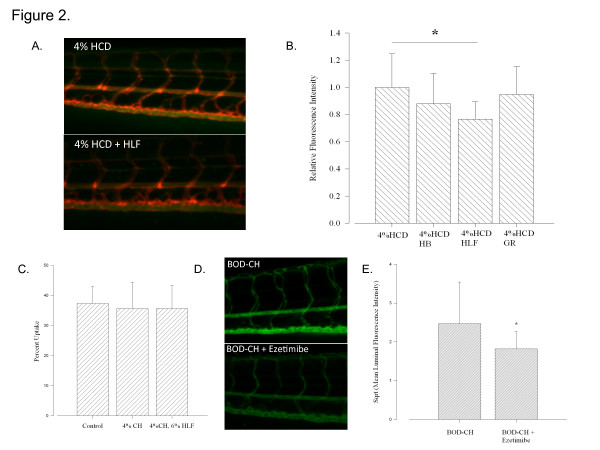
**Hawthorn Leaves and Flowers (HLF) Decrease BODIPY-cholesterol fluorescence in 4%HCD-fed fish. A**. Representative images of the vasculature of fish in 4%HCD and 4%HCD + HLF treated fish. Orange vasculature from *TG(Kdrl:mCherry)/Casper* line. Green is intravascular BOD-CH fluorescence **B**. Fluorescence output comparison between groups. Analyzed relative to mean of 4% HCD group having a value of 1. Significant difference between HLF and 4%HCD treated fish. * P < 0.05, bars are mean ± S.D. 1-way ANOVA, Holm-Sidak post-hoc multiple comparison. n = 15-17 per group. **C**. Comparison of percent uptake between untreated (control), CH-treated, and CH + HLF-treated diets. Five in each experimental group, with the experiment repeated 3x, showed similar uptake (P = 0.925, bars are mean ± S.D. of repeated experiments. 1-way ANOVA). **D**. Representative Images of BOD-CH treated and BOD-CH + Ezetimibe treated fish (4 dpf). **E**. Ezetimibe treatment decreases intravascular BOD-CH fluorescence (* P < 0.05, bars are mean ± S.D. 1-way ANOVA, Holm-Sidak post-hoc multiple comparison. n = 13-14 per group).

In order to test the response of hypercholesterolemic zebrafish to plant-based dietary intervention, we fed fish a diet containing phytomedicine and CH. We treated HCD-fed fish with HB, HLF and GR infused diets daily between 8–20 dpf. To each group we added equivalent amounts of BOD-CH. We found that HLF significantly decreased intravascular BOD-CH fluorescence levels compared to HCD-treated controls in our model (Figure2.). These results agree with the proposed antihypercholesterolemic effects of HLF in humans. To ensure the decreased fluorescence intensity due to HLF treatment was not due to decreased dietary uptake, we tested the percent uptake of regular, CH-treated and CH + HLF-treated diets. The uptake of the diets was nearly identical between these groups. Further, as a positive control to ensure BOD-CH fluorescence relates to CH level, we treated fish with the Neimann-Pick C1 Like-1 (NPC1L1) receptor inhibitor ezetimibe, a potent blocker of intestinal cholesterol absorption. This lead to decreased intravascular BOD-CH fluorescence (Figure2D and 2E), which implies that BOD-CH uptake is similar to cholesterol and further validates our model.

### A high cholesterol diet decreases cardiac output in the zebrafish model

**Figure 3:**
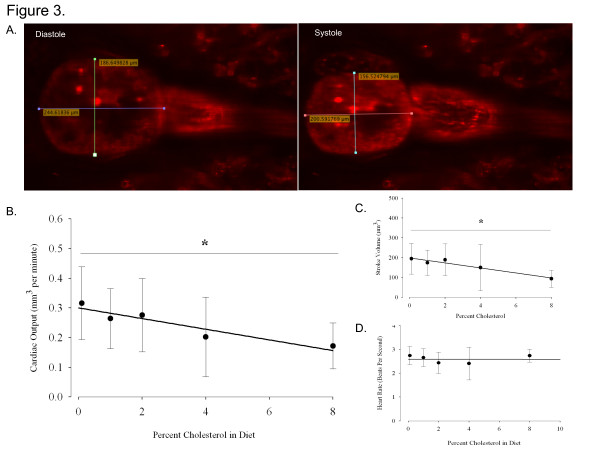
**Cardiac Output (CO) Decreases with Increasing Cholesterol (CH) Dose. A**. Measurement of ventricular diastole and systole in *TG(Kdrl:mCherry)/Casper* transgenic to derive stroke volume (SV) in order to finally calculate CO **B**. CO decreases with increasing CH dose from 0.1-8%HCD (R = 0.397, R^2^ = 0.158). Difference between 0.1% and 8%HCD effect on CO is significant C. SV also decreases with increasing CH dose from 0.1-8%HCD (R = 0.396, R^2^ = 0.157). Difference between 0.1% and 8%HCD effect on SV is significant C. There is not a relationship between HR and CH dose. * P < 0.05, points are mean ± S.D. 1-way ANOVA, Holm-Sidak post-hoc analysis. n = 12-13 per group.

The purpose of this experiment was to determine whether an HCD impairs cardiovascular performance in the fish, and in doing so elucidate an optimal CH dose to induce cardiac dysfunction. Cardiac output (CO) is an excellent measure of overall cardiovascular function, as its measurement provides information about volume flow throughout the cardiovascular system as well as cardiac dynamics. As expected, an increasing dose of CH correlated with decreased CO (Figure3A). Significantly impaired function was observed at 8% HCD compared to the lowest dose (0.1% HCD) (Figure3A). This decrease appears to have been due to a decreased stroke volume (SV) as evidenced by a similar relationship between SV and CO to percent CH in the diet (Figure3B). There was no apparent correlation between HR and percent CH (Figure3C). Taken together, this data indicates that the zebrafish develops significantly weakened cardiovascular function after 3 weeks of 8% HCD feeding. Therefore, our results provide evidence that the larval zebrafish can be utilized to test the effects of an HCD on cardiodynamic pathophysiology.

### Hawthorn leaves and flowers interact with cholesterol to affect cardiac output

**Figure 4:**
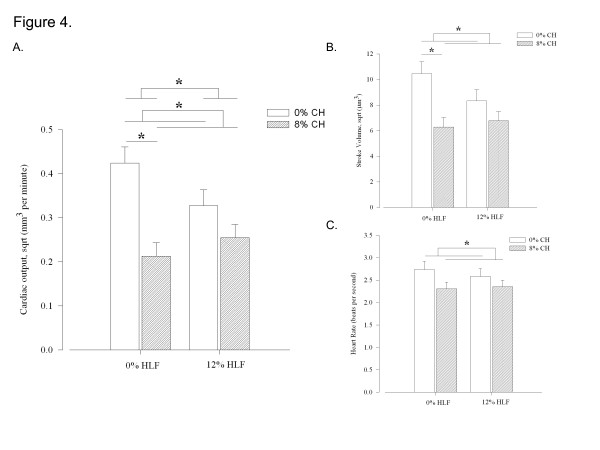
**Hawthorn leaves and flowers (HLF) interact with cholesterol (CH) to impact cardiac output (CO). A**. HLF show significant interaction with CH in their influence on CO. CH decreases CO within 0% HLF and CH decreases CO when comparing all 0% CH-treated to all 8% CH-treated fish **B**. No interaction effect was observed on stroke volume (SV). CH decreases SV within 0% HLF and CH decreases SV when comparing all 0% CH-treated to all 8% CH-treated fish **C**. No interaction effect was observed on heart rate (HR). CH decreases HR when comparing all 0% CH-treated to all 8% CH-treated fish. * P < 0.05, bars are mean + S.D. 2-way ANOVA with Holm-Sidak post-hoc analysis. n = 12-19 per group.

The ability of HLF to decrease intravascular CH levels indicates that it is a potential treatment for hypercholesterolemia, but its impact on the peripheral pathology of this disease is unknown. We therefore used our model to test the ability of HLF to impact HCD-induced cardiac pathophysiology. Two-way ANOVA analysis revealed a significant interaction between HLF and CH in their effects on CO (Figure4A). CH and HLF do not show significant interaction in their effects on SV or HR (Figure4B and 4C). Therefore, the interaction observed between HLF and CH in affecting CO can be explained by the combined influence of both SV and HR.

Supporting this contention, with this more detailed analysis, we were able to detect a significant effect of CH on both SV and HR (Figure4B and 4C).

## Discussion

One goal of this study was to develop the larval zebrafish as a model to test the efficacy of the numerous potential phytotherapeutic treatments of hypercholesterolemia. The other purpose of our work was to exercise a holistic perspective to the treatment and quantification of disease that is relatable to the traditional philosophies prescribing these medicines.

Our initial experiments focused on finding a fluorescent marker for dietary CH intake. This was so we could later test the ability of selected whole phytomedicines to treat hypercholesterolemia. Utilizing BOD-CH, we were able to see a significant difference in intravascular fluorescence between HCD-treated and control fish that we did not see in the NBD-treated group. The accumulation of sterol-based fluorophore deposits in the vasculature of HCD-fed zebrafish was first observed in [4] with the probe cholesteryl-BODIPY. In our experiments with BOD-CH, we also observed more fluorescent deposit formation in the vasculature of HCD-fed, BOD-CH treated fish than in controls (Figure1A), however, the formation of these deposits was extremely variable between individuals. In the NBD-CH treated group we saw a large amount of fluorescent deposit accumulation in the vascular endothelium of both control and treated groups (Figure1A). These results indicate that an HCD did not influence the formation of these deposits in NBD-treated fish (Figure1A). A potential reason for this is that NBD-CH displays properties more similar to the oxysterol, 25-hydroxycholesterol, than it does to CH itself [26]. Therefore, 22-NBD-cholesterol may not be physiologically processed similarly to cholesterol. Supporting this argument, Adams *et al.*[27] demonstrated that NBD-CH is absorbed by an ezetimibe-insensitive and Neimann-Pick C1 Like-1 (NPC1L1) protein independent pathway. This indicates that the intestinal absorption of NBD-CH is different from native CH as ezetimibe acts to block the intestinal absorption of CH by inhibiting NPC1L1. More work needs to be done to confirm the metabolism and localization of BODIPY-cholesterol in the zebrafish is similar to native CH, but our results and the work of others indicate that it is likely comparable [28,29].

Utilizing our methodology, we found that HLF significantly reduced intravascular BOD-CH levels in HCD-fed fish. There was a slight but not significant decrease in BODIPY fluorescence in the HB treated group. Therefore, it is likely that berries are a less potent CH reducing treatment than HLF. This information may help to identify a class of compounds in *Crataegus laevigata* responsible for decreasing CH levels. HLF and HB have many potentially active ingredients in common, including procyanidin and flavonoid components. All three treatments tested (HLF, HB and GD) contain flavonoids [30,31]. It is therefore unlikely that the flavonoid components are affecting vascular CH levels. HLF (*Crataegus laevigata*) contain 1.2-1.6% procyanidin content whereas HB contains 0.2% procyanidin [30]. There is no data to our knowledge suggesting that GR contains procyanidins, this difference in procyanidin content could account for the reduced ability of HB to impact CH levels compared to HLF. It also explains why GR failed to impact intravascular BOD-CH fluorescence.

Our holistic approach yielded a surprising result: While the combination of CH/HLF slightly improved CO compared to CH alone, fish treated with either CH or HLF alone exhibited decreased CO. In essence, there was no combined effect between CH and HLF to decrease CO; rather CH and HLF together resulted in improved CO compared to CH treatment alone. This result illustrates the benefit of a holistic approach to treatment where there is a strong potential for unanticipated effects. In order to explain these results however, it is necessary to apply a reductionist lens to our analysis. Long *et al.*[32] found that some fractions of *Crataegus oxycantha* leaves and flowers have a negative chronotropic effect on isolated cardiomyocytes while others have a positive chronotropic effect*.* This indicates that different components of hawthorn can have contradictory effects, suggesting that if the balance between components is altered, the effects of the treatment can be changed. In our experiment, two-way ANOVA analysis revealed a significant interaction between CH and HLF in their effects on CO. We hypothesize that when zebrafish were fed CH/HLF, negatively inotropic components of HLF sequestered CH. More specifically, procyanidin components of HLF blocked intestinal absorption of CH and therefore could not affect CO. The balance of positively and negatively inotropic components was therefore altered toward increasing the availability of positive inotropic components. Combined with a reduction in cholesterol levels, this increase of available positive inotropic plant constituents lead to the CO increase in CH/HLF treated fish compared to CH-treated.

This array of potential effects and treatment paradigms from which to view a single phytomedicine demonstrates the need for experimenting with a large number of organisms. It would therefore be beneficial to have a high throughput method capable of assessing the ability of these phytomedicines to treat HCD-induced cardiovascular disease. The zebrafish model organism provides an excellent opportunity to achieve this, as zebrafish-based high-throughput screening methods capable of screening for cardiovascular disease are a burgeoning method of drug discovery. With high-throughput screening, it will be possible to test compound classes to derive the synergistic effects of the plant parts and which ratio of, for example, procyanidin to flavonoid, best treats a particular disease.

## Conclusions

The results of this study demonstrate that HLF are a promising treatment for hypercholesterolemia. We have created a vertebrate model system to test plant-based dietary intervention of hypercholesterolemia using the zebrafish. We utilized this model to demonstrate that HLF decrease intravascular CH levels and interact with cholesterol to improve CO in diseased fish. These results indicate that the cardiotonic action of hawthorn may depend on diet. Further work is needed to understand the detailed mechanism of how HLF exerts its influence. While hawthorn is not currently a recommended treatment for hypercholesterolemia, our research indicates it may be an effective treatment for this disease.

## Competing interests

The author(s) declare that they have no competing interests.

## Authors' contributions

RML conceived of this study, designed and performed the experiments, carried out all data and statistical analyses and was the primary author of this manuscript. MM assisted with experiments and provided meaningful insight. JRH assisted in the conception of this study, the design of the experiments and in preparing this manuscript. All authors read and approved the final manuscript.

## Pre-publication history

The pre-publication history for this paper can be accessed here:

http://www.biomedcentral.com/1472-6882/12/105/prepub

## Supplementary Material

Additional file 1 **Figure S1. Zebrafish Length Assessment: 0% *vs.* 4% High Cholesterol Diet (HCD).** Comparison of zebrafish length between control and 4% HCD showing similar length between treatment groups. Click here for file
