# Supplementary material for: Whole plant based treatment of hypercholesterolemia with Crataegus laevigata in a zebrafish model
Source: BMC Complement Altern Med. 2012 Jul 23;12:105. doi: 10.1186/1472-6882-12-105 (PMC3479075; doi:10.1186/1472-6882-12-105)
Supplement: Additional file 1 — Figure S1. Zebrafish Length Assessment: 0% vs. 4% High Cholesterol Diet (HCD). Comparison of zebrafish length between control and 4% HCD showing similar length between treatment groups. [file 1472-6882-12-105-S1.pdf]

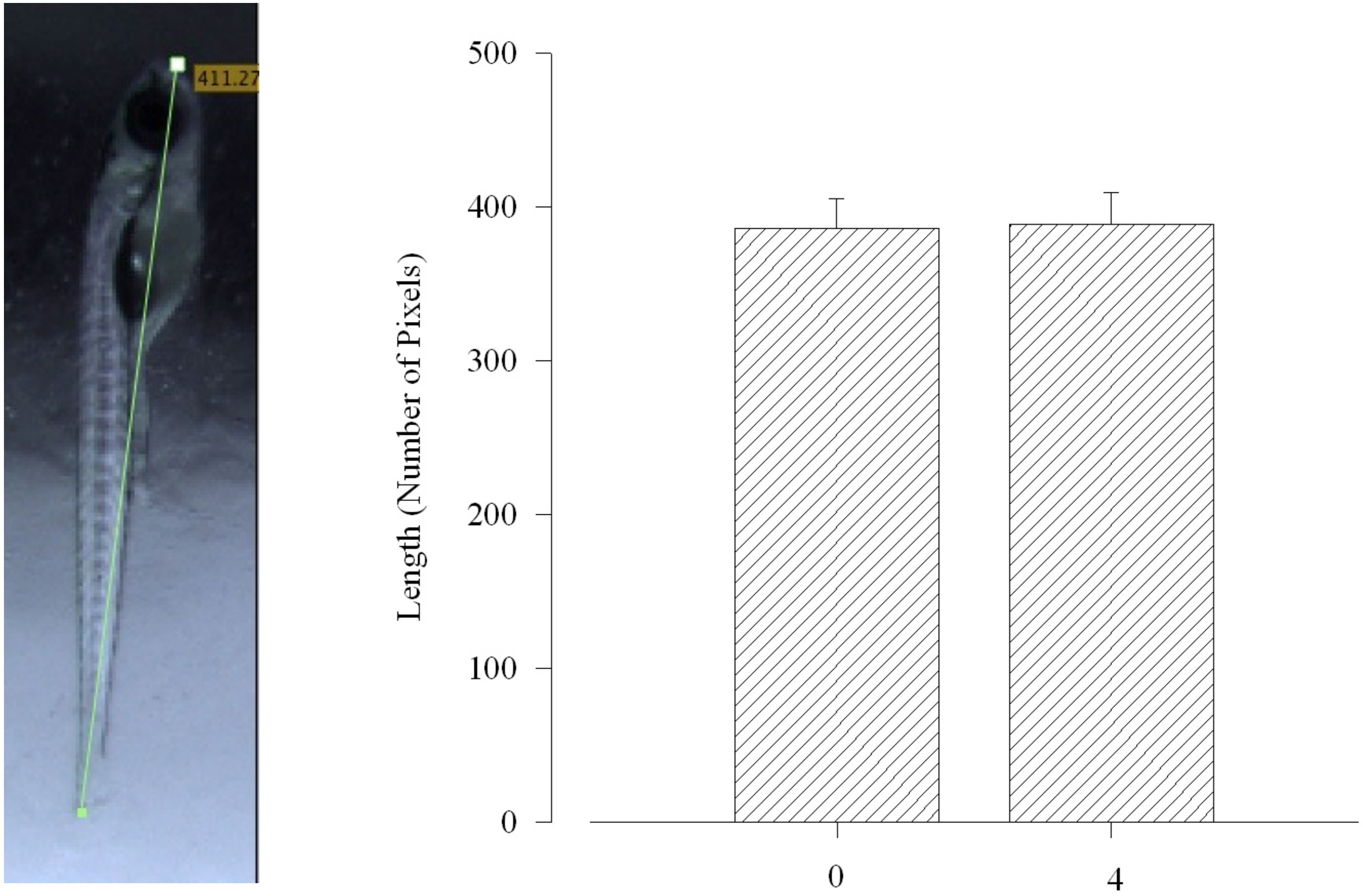

Supplemental Figure. A. Image of 18 dpf zebrafish with length measured in number of pixels. B. No difference detected between the length of 0% CH and 4% CH diet fed fish (n=8 individuals per group. P = 0.769. Student's t-test).
